# The efficacy of repetitive transcranial magnetic stimulation in postherpetic neuralgia: a meta-analysis of randomized controlled trials

**DOI:** 10.3389/fneur.2024.1365445

**Published:** 2024-06-11

**Authors:** Qi Dai, Aihua Xu, Kunpeng Wang, Yang Yang, Yang Shao, Yongxin Sun

**Affiliations:** ^1^Department of Rehabilitation Medicine, The First Hospital of China Medical University, Shenyang, China; ^2^Department of Pain Medicine, The First Affiliated Hospital, China Medical University, Shenyang, China

**Keywords:** repetitive transcranial magnetic stimulation, postherpetic neuralgia, meta-analysis, efficacy, systematic review

## Abstract

**Purpose:**

This systematic review and meta-analysis aimed to evaluate the efficacy of repetitive transcranial magnetic stimulation (rTMS) in postherpetic neuralgia (PHN).

**Methods:**

Through an extensive search in four databases until October 2023, we selected five randomized controlled trials adhering to our specific criteria, involving 257 patients in total. For continuous outcomes, the standardized mean difference (SMD) was calculated. Heterogeneity among the studies was assessed using Cochran’s *I*^2^ and *Q* statistics, adopting a random-effects model for *I*^2^ values over 50%. For assessing potential publication bias, we utilized both funnel plot and Egger’s test.

**Results:**

Our analysis found that rTMS reduced the overall visual analogue scale (VAS) (SMD: −1.52, 95% CI: −2.81 to −0.23, *p* = 0.02), VAS at 1 month post-treatment (SMD: −2.21, 95% CI: −4.31 to −0.10, *p* = 0.04), VAS at 3 months post-treatment (SMD: −1.51, 95% CI: −2.81 to −0.22, *p* = 0.02), as well as patients’ global impression of change scale (PGIC) (SMD: −1.48, 95% CI: −2.87 to −0.09, *p* = 0.04) and short-form McGill pain questionnaire (SF-MPQ) (SMD: −1.25, 95% CI: −2.41 to −0.09, *p* = 0.03) compared to the sham-rTMS group.

**Conclusion:**

Our study suggests that rTMS might have a potential alleviating effect on PHN symptoms. However, due to the limited number of studies and variations in rTMS parameters, larger sample studies involving more diverse populations, as well as further clarification of the most appropriate stimulation protocol, are still needed.

**Systematic review registration:**

https://www.crd.york.ac.uk/prospero/, Identifier ID: CRD42023488420.

## Introduction

Postherpetic neuralgia (PHN) is a common complication of herpes zoster. It generally refers to pain that persists for more than 1 month after the healing of herpes zoster rash (1), and is also defined as pain lasting more than 90 days (2). The incidence rate of herpes zoster is 10 to 20%, with 9 to 13% of patients developing PHN (3), and the incidence of this disease has been gradually increasing in recent years (4). The pain of PHN is characterized by spontaneous pain, pain hypersensitivity, allodynia, and abnormal sensations, with a long-lasting course, leading to negative emotions like anxiety, despair, and depression in patients, significantly affecting their quality of life (1, 5). Early and effective treatment is very important for PHN patients, as it can have a profound impact on their quality of life, including physical, emotional, and social aspects.

Currently, pharmacotherapy remains the primary method for treating PHN. However, the effectiveness of pharmacotherapy is not satisfactory. For most patients, medication only partially relieves pain. This is mainly because the adverse reactions of the drugs limit the achievable doses (6). Other measures, such as botulinum toxin, nerve blocks, spinal cord stimulation, and radiofrequency, also make significant contributions to the treatment of PHN. However, these treatments are invasive, and their effectiveness, safety, and tolerability still need to be monitored (7). Considering the limitations of the current treatments, seeking alternative, effective, and safer therapeutic methods is of great importance.

Transcranial magnetic stimulation (TMS) is based on the principle of electromagnetic induction, utilizing the magnetic field pulses generated when a strong varying current passes through a coil placed over the head, and the resulting induced current that initiates action potentials (8), affecting the entire brain functional network (9). Repetitive transcranial magnetic stimulation (rTMS), as a non-invasive brain stimulation technique, has emerged as a promising intervention in the treatment of neuropathic pain (10). However, there is limited research on rTMS for treating PHN, and there is controversy over its effectiveness (11–16). The improvement of short-form McGill pain questionnaire (SF-MPQ) (11, 14, 15, 17) and patients’ global impression of change scale (PGIC) (14, 15, 17) post-treatment is also a subject of debate.

Therefore, it is necessary to summarize and analyze the data from published studies. The purpose of this meta-analysis is to integrate the current evidence from randomized controlled trials to evaluate the efficacy of rTMS in the treatment of PHN.

## Materials and methods

The meta-analysis rigorously followed the Preferred Reporting Items for Systematic Reviews and Meta-analyses (PRISMA) 2020 guidelines. We registered the protocol for this meta-analysis with PROSPERO under the identifier CRD42023488420.

### Search strategy

A comprehensive search was conducted in databases including PubMed, Embase, China National Knowledge Infrastructure (CNKI), and WANFANG DATA, for relevant publications up to October 2023. Search terms are listed in the Supplementary Table S1. Additionally, we manually examined the reference lists of selected articles to identify additional relevant research.

### Inclusion and exclusion criteria

This meta-analysis includes randomized controlled trials evaluating the efficacy of rTMS in PHN, requiring a minimum sample size of 10 participants.

The exclusion criteria covered duplicate articles, letters, case reports, reviews, meta-analyses, and irrelevant titles or abstracts. Studies offering incomplete or equivocal data, impeding accurate outcome assessment, were also excluded. Furthermore, we only included studies in English in patient overlap situation (articles with patient overlap that have both Chinese and English versions).

Two researchers independently evaluated article titles and abstracts based on predefined inclusion and exclusion criteria. Following this, they thoroughly inspected the full text to ascertain the studies’ eligibility. Disagreements were addressed and resolved by discussion, leading to a consensus.

### Quality assessment

Using the Cochrane Risk of Bias Tool for randomized trials, a pair of independent researchers assessed the quality levels of the selected studies. Factors such as random sequence generation (selection bias), allocation concealment (selection bias), blinding of participants and personnel (performance bias), blinding of outcome assessment (detection bias), incomplete data (attrition bias), selective reporting (reporting bias), and other biases were examined by both reviewers. If there were any discrepancies, a third researcher would be consulted to resolve the issue.

### Data extraction

Two researchers independently extracted data from each included article, including author, year, country, study design, outcome, comparison, mean age ± SD, male/female, number of patients, pain laterality (right), as well as parameters and dosage. The disagreements among researchers were settled through discussions, eventually leading to a consensus.

### Outcome measures

The primary outcome was the overall visual analogue scale (VAS) score, a straightforward tool for pain assessment. Increased VAS scores denote higher levels of pain (18, 19). The secondary outcome measures also include VAS at 1 month post-treatment, VAS at 3 months post-treatment, PGIC, and SF-MPQ. PGIC indicates the comprehensive changes in pain relief, functional improvement, emotional state, and quality of life after therapeutic interventions, also eliminating the misconception that pain reduction alone indicates successful treatment. The lower the PGIC score, the more effective the treatment is deemed (20, 21). SF-MPQ is a sensitive and dependable pain assessment tool that not only evaluates the characteristics of pain but also precisely measures the patient’s emotional and sensory experiences, encompassing fatigue, discomfort, fear, and torment. The higher the SF-MPQ score, the more severe the pain, and the worse the emotional and sensory experiences (15, 20).

### Statistical analysis

In this analysis, we utilized the standardized mean difference (SMD) to evaluate continuous outcomes. Corresponding 95% confidence intervals (CIs) were also calculated to estimate the range of the effect sizes. Heterogeneity among the studies was assessed using Cochran’s *I*^2^ and *Q* statistics. Heterogeneity, based on *I*^2^ values, was classified as low (25%), moderate (50%), or high (75%). A fixed-effects model was applied for *I*^2^ values below 50%, above this threshold, a random-effects model was used. In instances of significant heterogeneity (*I*^2^ ≥ 50%), leave-one-out sensitivity analyses were performed to find out the heterogeneity sources and assess the stability of the results.

Publication bias, which is the tendency to favor publishing studies with positive or significant outcomes, was assessed through funnel plot analysis and Egger’s test. Analyses were performed using Stata 17 software.

## Results

### Literature search and study selection

**Figure 1:**
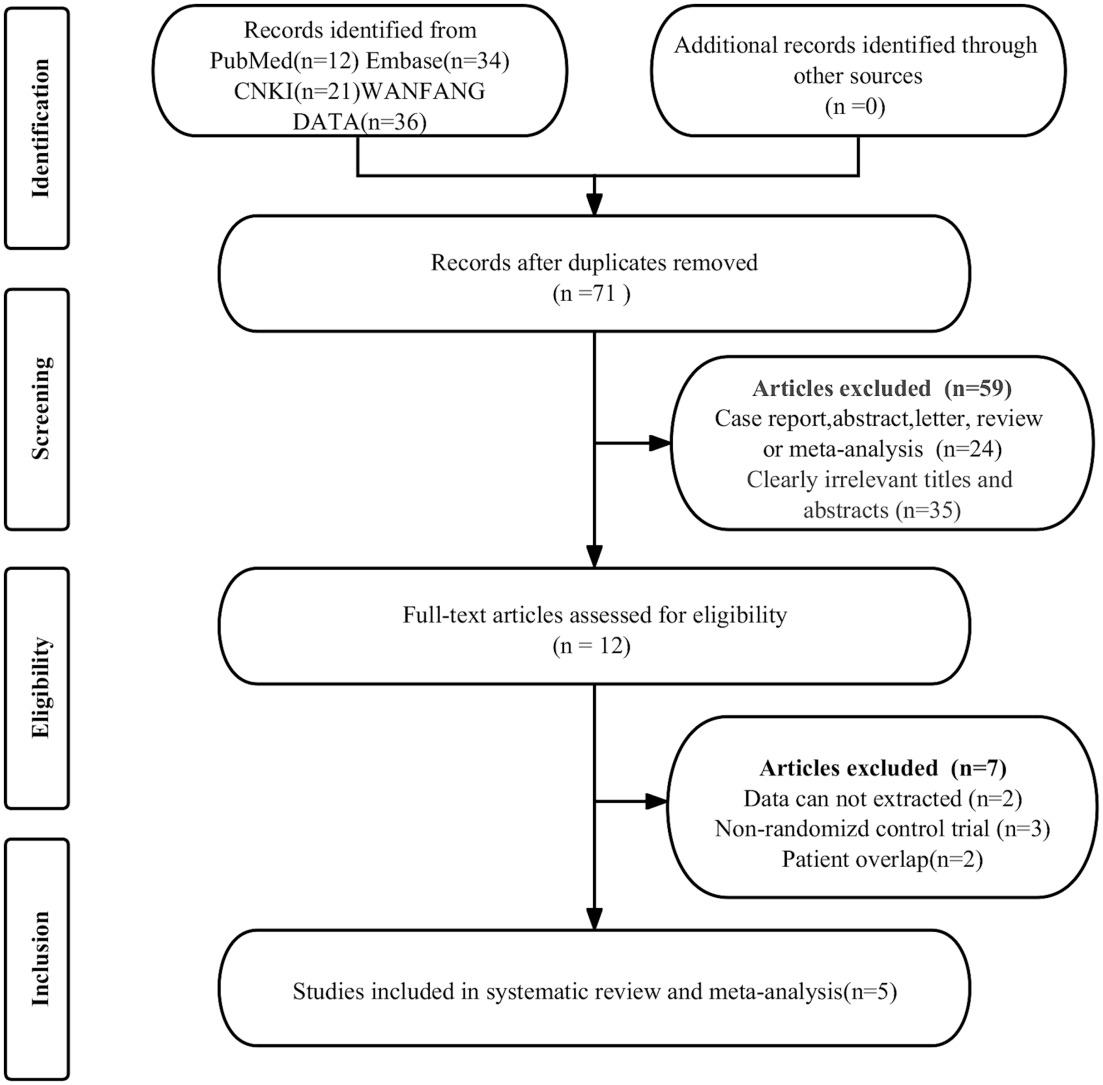
PRISMA flow diagram illustrating the study selection process. PRISMA, preferred reportingitems for systematic reviews and meta-analyses.

The initial search identified 103 publications, of which 32 were duplicates and 59 were ineligible, leading to their exclusion. Further scrutiny of the full texts of the remaining 12 articles led to the exclusion of 7 more studies due to dates that could not be extracted (*n* = 2), non-randomized control trials (*n* = 3) and patient overlap (*n* = 2). This process resulted in the selection of 5 randomized controlled trials for the analysis of the efficacy of rTMS in PHN (11, 14–17). Figure 1 displays the PRISMA flow diagram depicting this selection process.

### Study description and quality assessment

In these eligible studies, five randomized controlled trials from China, encompassing a total of 257 patients, were evaluated. Their characteristics are succinctly summarized in Table 1. The patient count in each study varied from 33 to 64, with their mean age ranging from 61.3 to 70.62 years. The stimulation frequency was 5 Hz or 10 Hz. The stimulation site was either the motor cortex (M1) or the dorsolateral prefrontal cortex (DLPFC). The intensity of stimulation was 80–100% motor threshold. Patients were treated for 10 or 15 sessions and each rTMS session delivered 1,200 to 3,000 pulses with intervals of 2.5 s, 3 s, or 25 s.

**Table 1:** The study characteristics of the included studies.

Author	Year	Country	Study design	Outcome	Comparison	Mean age ± SD	Male/Female	Number of patients	Pain laterality (right)	Parameters and Dosage
Wang et al.	2023	China	Randomized controlled trial	(1) (3)	rTMS	69.65 ± 8.60	26/14	40	18	M1/DLPFC, 10 Hz, 100%MT, 50pulses × 60trains/session, 10sessions days, 25 s-intervals
					Sham rTMS	67.05 ± 7.67	7/13	20	4	NA
Pei et al.	2019	China	Randomized controlled trial	(1) (2) (3)	rTMS	65.65 ± 11.29	19/21	40	14	M1, 5 Hz/10 Hz, 80%MT, 5pulses × 300trains/session,10sessions days, 2.5 s/3 s-intervals
					Sham rTMS	67.3 ± 11.9	11/9	20	7	NA
Ma et al.	2015	China	Randomized controlled trial	(1) (2) (3)	rTMS	65.4 ± 10.5	11/9	20	7	M1, 10 Hz, 80%MT, 5pulses × 300trains/session, 10sessions days, 3 s-intervals
					Sham rTMS	67.3 ± 11.9	9/11	20	7	NA
Pu et al.	2017	China	Randomized controlled trial	(1) (2) (3)	rTMS	70.62 ± 8.55	9/12	21	12	M1, 10 Hz, 80–100%MT, 1200 pulses, 15sessions days
					Sham rTMS	66.58 ± 8.26	8/4	12	5	NA
Chen et al.	2021	China	Randomized controlled trial	(1)	rTMS	62.7 ± 5.8	16/16	32	NA	M1, 10 Hz, 80%MT, 5pulses × 300trains/session, 10sessions days, 3 s-intervals
					Sham rTMS	61.3 ± 4.9	19/13	32	NA	NA

**Figure 2:**
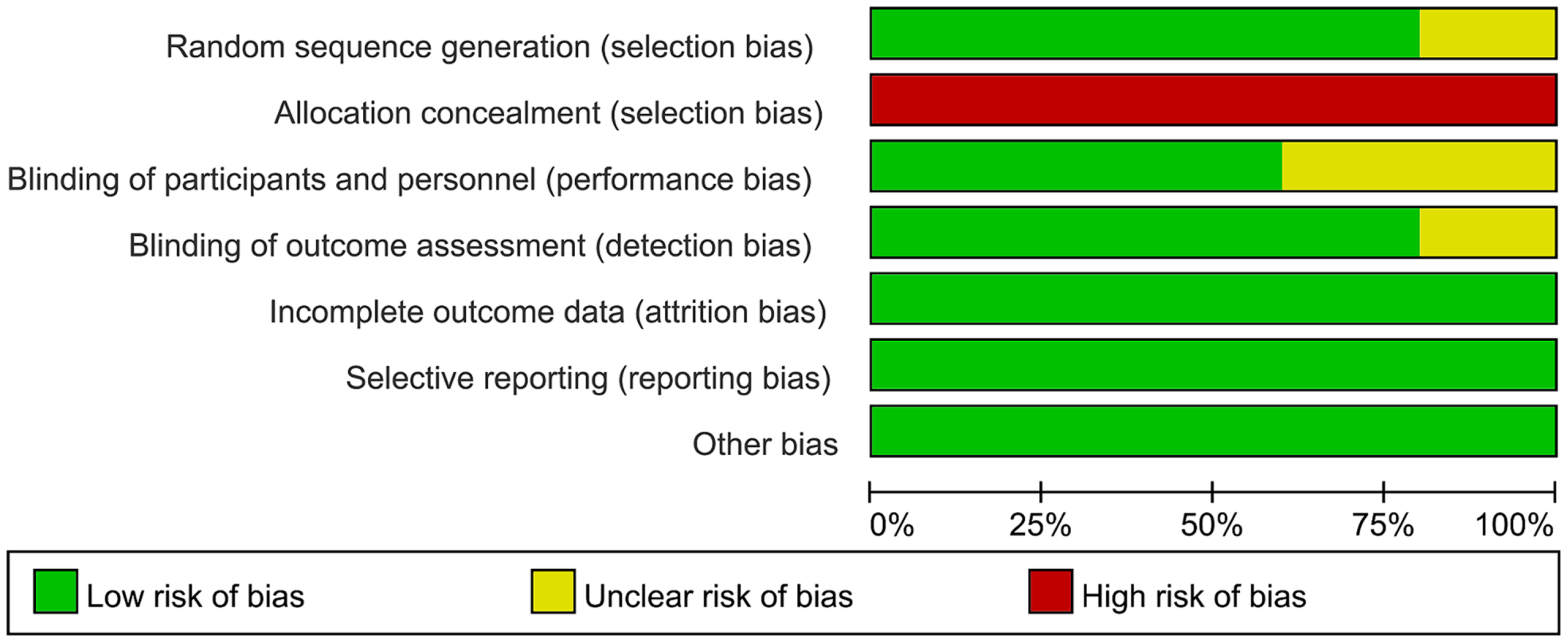
Risk of bias of the included studies using the Cochrane risk of bias tool for randomized trials.

Figure 2 illustrates the risk of bias in each study, as assessed using the Cochrane Risk of Bias Tool. High risk is primarily concentrated in allocation concealment (selection bias), as the operators administering rTMS treatment must know the allocation plan to provide the corresponding treatment to patients. Overall, the included studies displayed acceptable quality.

### Quantitative analysis of overall VAS

**Figure 3:**
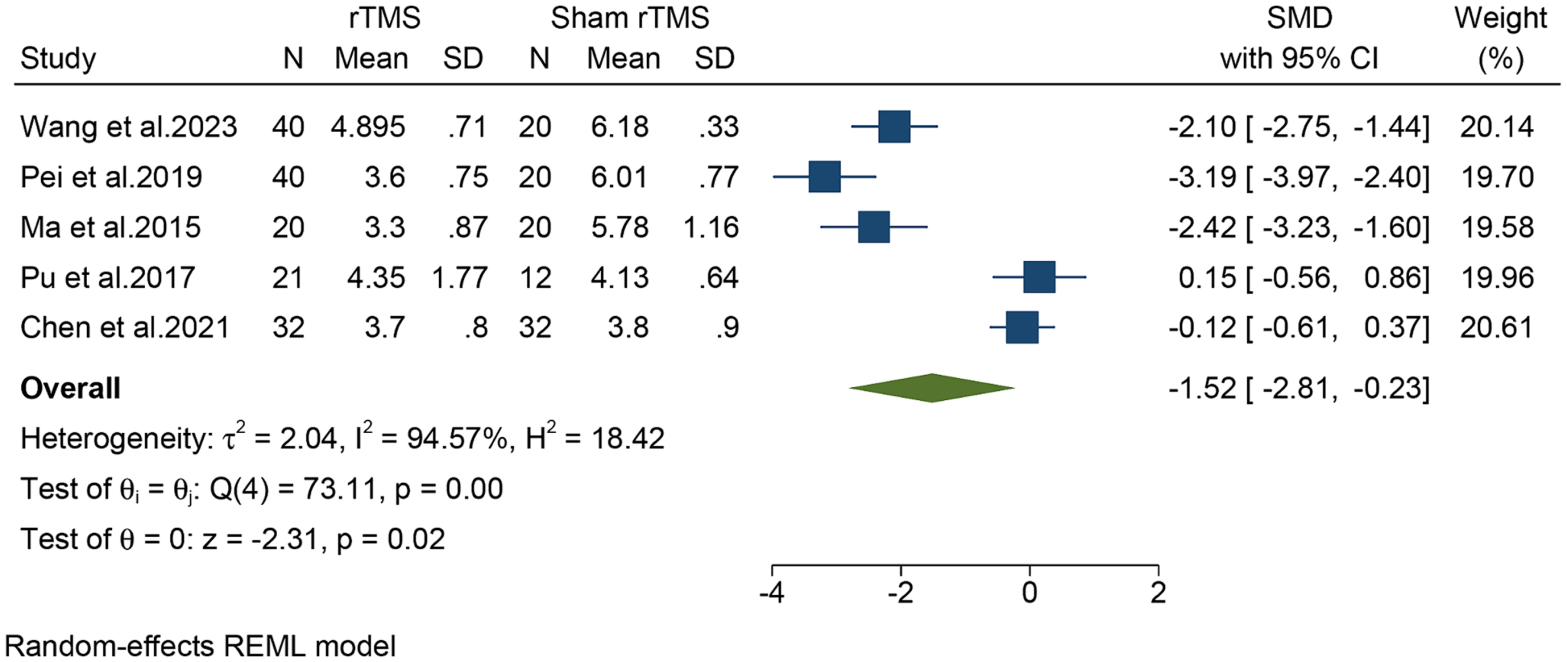
Forest plot of the overall VAS for the rTMS group vs. the Sham rTMS group. VAS, visual analogue scale; rTMS, repetitive transcranial magnetic stimulation.

**Figure 4:**
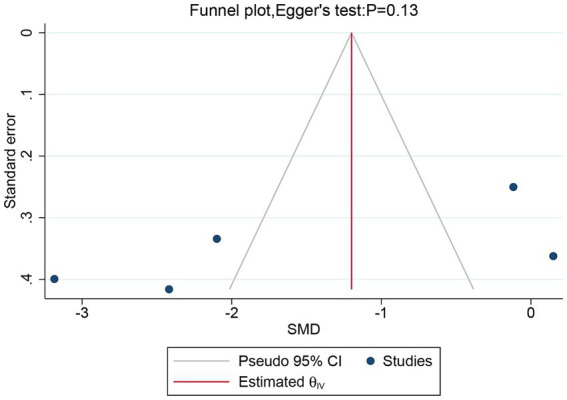
Funnel plot and Egger’s test of the overall VAS for the rTMS group vs. the Sham rTMS group. VAS, visual analogue scale; rTMS, repetitive transcranial magnetic stimulation.

Five studies, including a total of 257 patients, evaluated the overall VAS. In light of the high heterogeneity (*I*^2^ = 94.57%, *p* = 0.02), we applied a random-effect model. The meta-analysis revealed that rTMS significantly decreased the overall VAS (SMD: −1.52, 95% CI: −2.81 to −0.23, *p* = 0.02) compared to the sham rTMS group (Figure 3). The sensitivity analysis found no potential sources of heterogeneity (Supplementary Table S2). There was no detectable publication bias as indicated by both funnel plot and Egger’s test (*p* = 0.13) (Figure 4).

**Figure 5:**
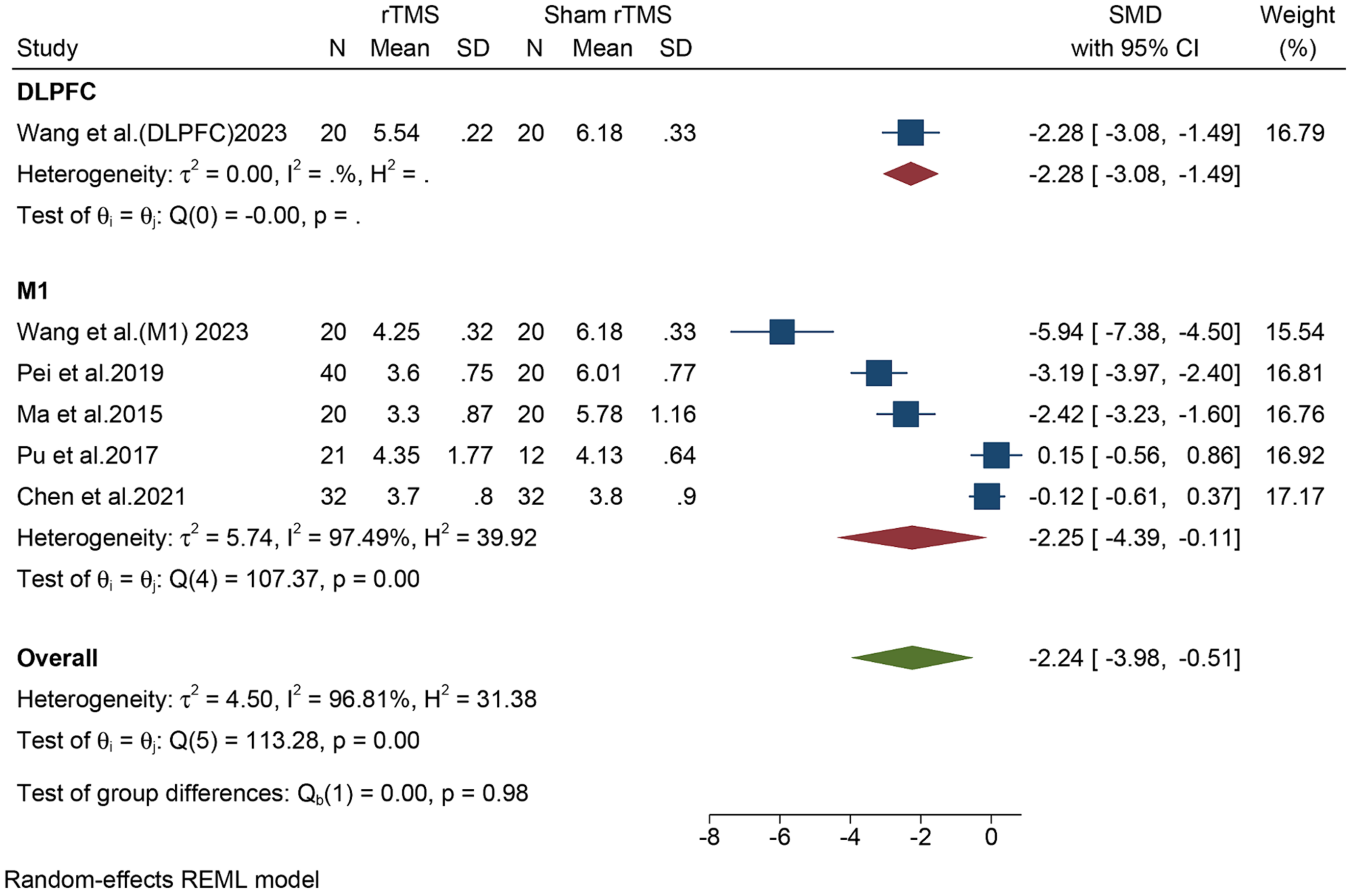
Subgroup analysis in different stimulate regions (M1 or DLPFC) of the overall VAS for the rTMS group vs. the Sham rTMS group. M1, motor cortex; DLPFC, dorsolateral prefrontal cortex; VAS, visual analogue scale; rTMS, repetitive transcranial magnetic stimulation.

Subgroup analyses in different regions (DLPFC vs. M1) were conducted. For DLPFC, one study, including a total of 40 patients, evaluated the overall VAS. The meta-analysis revealed that rTMS significantly decreased the overall VAS (SMD: −2.28, 95% CI: −3.08 to −1.49) compared to the sham rTMS group (Figure 5). For M1, five studies, including a total of 237 patients, evaluated the overall VAS. In light of the high heterogeneity (*I*^2^ = 97.49%, *p* = 0.00), we applied a random-effect model. The meta-analysis revealed that rTMS also significantly decreased the overall VAS (SMD: −2.25, 95% CI: −4.39 to −0.11) compared to the sham rTMS group (Figure 5). Although both DLPFC and M1 can reduce the overall VAS, there was no statistical difference between the two groups (*p* = 0.98).

### Quantitative analysis of VAS at 1 month post-treatment

Five studies, including a total of 257 patients, evaluated the VAS at 1 month post-treatment. In light of the high heterogeneity (*I*^2^ = 97.69%, *p* = 0.04), we applied a random-effect model. The meta-analysis revealed that rTMS significantly decreased the VAS at 1 month post-treatment (SMD: −2.21, 95% CI: −4.31 to −0.10, *p* = 0.04) compared to the sham rTMS group (Supplementary Figure S1). The sensitivity analysis found no potential sources of heterogeneity (Supplementary Table S3).

### Quantitative analysis of VAS at 3 months post-treatment

Five studies, including a total of 257 patients, evaluated the VAS at 3 months post-treatment. In light of the high heterogeneity (*I*^2^ = 94.63%, *p* = 0.02), we applied a random-effect model. The meta-analysis revealed that rTMS significantly decreased the VAS at 3 months post-treatment (SMD: −1.51, 95% CI: −2.81 to −0.22, *p* = 0.02) compared to the sham rTMS group (Supplementary Figure S2). The sensitivity analysis found no potential sources of heterogeneity (Supplementary Table S4).

### Quantitative analysis of PGIC

Three studies, including a total of 133 patients, evaluated the PGIC. In light of the high heterogeneity (*I*^2^ = 91.27%, *p* = 0.04), we applied a random-effect model. The meta-analysis revealed that rTMS significantly decreased the PGIC (SMD: −1.48, 95% CI: −2.87 to −0.09, *p* = 0.04) compared to the sham rTMS group (Supplementary Figure S3). The sensitivity analysis found no potential sources of heterogeneity (Supplementary Table S5).

### Quantitative analysis of SF-MPQ

Four studies, including a total of 193 patients, evaluated the SF-MPQ. In light of the high heterogeneity (*I*^2^ = 91.63%, *p* = 0.03), we applied a random-effect model. The meta-analysis revealed that rTMS significantly decreased the SF-MPQ (SMD: −1.25, 95% CI: −2.41 to −0.09, *p* = 0.03) compared to the sham rTMS group (Supplementary Figure S4). The sensitivity analysis found no potential sources of heterogeneity (Supplementary Table S6).

## Discussion

Our meta-analysis indicates that compared to the sham rTMS group, rTMS significantly reduced the VAS scores in PHN patients, thereby alleviating the pain. At present, the pain-relieving mechanism of rTMS is unclear. There are some studies that suggest rTMS of the motor cortex may activate brain regions associated with descending pain modulation (22–25). This pattern of distant and diffuse brain activation is consistent with the diffuse and non-somatotopic analgesic effect induced by rTMS in the motor cortex, as many studies have shown, where localized stimulation leads to pain reduction in different body areas (26–28). Additionally, Goto et al. (29) observed that the integrity of the corticospinal tract and thalamocortical tract is significant for the pain alleviation induced by rTMS. rTMS can also regulate local cerebral blood flow and metabolism (30), and promote the release of cerebral beta-endorphin, recognized as a pain-relieving factor in the nervous system (31). Additionally, studies have shown that enhancing neuroplasticity (32), reducing the levels of neuronal nitric oxide synthase overexpressed in dorsal root ganglia, and inhibiting astrocyte activity (33) could be the pain-relieving mechanisms of rTMS.

It is noteworthy that our study observed that rTMS in both the DLPFC and M1 regions led to a significant reduction in VAS, but no statistical difference was detected between them (*p* = 0.98). This finding differs from Wang et al. (11), who suggested that stimulating the M1 region has a more pronounced analgesic effect. This discrepancy may be due to the smaller number of patients included in the M1 group in their experiment, as they only included 20 patients in the M1 group compared to the DLPFC group. However, due to the overall limited number of studies on DLPFC stimulation, further research is still needed. Additionally, based on multiple studies, M1 remains the most commonly used stimulation target.

Furthermore, our analysis also shows that VAS scores significantly decreased at both 1- and 3 months post-treatment, indicating that the therapeutic effect of rTMS is lasting and stable. The improvements in PGIC and SF-MPQ further confirm the treatment’s effectiveness from the viewpoint of patient-centered care.

Jiang et al. (34) included a broader range of neuropathic pain cases in their meta-analysis, demonstrating a significant benefit of rTMS over sham rTMS. Although their results are consistent with the direction of our study, they did not specifically focus on PHN patients as our study did. Our study is the first to exclusively include PHN patients, providing a more targeted assessment of the efficacy of rTMS in this group. Additionally, our study’s inclusion of PGIC and SF-MPQ assessments offers a more comprehensive view of patient outcomes, a dimension that Jiang et al. did not explore. In another meta-analysis, Jin et al. (35), found that high-frequency rTMS stimulation of the primary motor cortex can effectively alleviate neuropathic pain. They primarily focused on the optimal treatment parameters of rTMS, including stimulation frequency and number of treatments, which differ from our outcome measures.

In the current meta-analysis, significant heterogeneity was observed in overall VAS, VAS at 1- or 3 months post-treatment, PGIC, and SF-MPQ, but the sensitivity analysis was unable to clarify the exact sources of this heterogeneity. This high level of heterogeneity might be attributed to differences in patient groups, research designs, or stimulation parameters in various studies. Future studies should aim to reduce these differences to better evaluate the effects of rTMS on treatment efficacy and to more effectively devise rTMS treatment plans. Furthermore, considering publication bias, we performed funnel plot and Egger’s test on the primary outcome measure, overall VAS, and detected no publication bias.

rTMS stands out for its rapid analgesic effects, non-invasiveness, and minimal side effects, being recognized as one of the significant brain science technologies of the 21st century (36, 37). Under strict adherence to treatment indications, the adverse effects are mainly headaches, occasional hearing loss, and very rarely, the induction of seizures. The treatment has a high safety profile (38). For elderly patients and those with severe comorbidities, cautious use of pharmacotherapy for PHN is advised, making rTMS treatment even more crucial (7). However, patients must undergo rTMS treatment in medical institutions, and the lack of standardized treatment protocols poses a challenge at present (39). Especially compared to traditional treatments, further cost-benefit research is needed in the future. Clinicians should weigh these factors and the individual circumstances of the patient when considering rTMS treatment for PHN.

Currently, our research still has limitations. The first limitation is that all study patients were from China. This means we cannot assume rTMS works the same for other races. More studies with different racial groups are needed. Secondly, despite conducting sensitivity analyses, the sources of heterogeneity were not identified, suggesting that the overall estimate effects of the meta-analysis should be interpreted with caution. At the same time, we observe that due to the studies originating from different institutions, there are variations in the parameters used for rTMS. Consequently, the most appropriate stimulation protocol remains to be clearly defined. Furthermore, the relatively small sample size of rTMS in PHN studies may affect the robustness of our conclusions. We look forward to future research, preferably with larger sample sizes and more diverse patient populations, to strengthen the evidence base for rTMS in the treatment of PHN.

## Conclusion

Our study suggests that rTMS might have a potential alleviating effect on PHN symptoms. However, due to the limited number of studies and variations in rTMS parameters, larger sample studies involving more diverse populations, as well as further clarification of the most appropriate stimulation protocol, are still needed.

## Data availability statement

The original contributions presented in the study are included in the article/Supplementary material, further inquiries can be directed to the corresponding author.

## Author contributions

QD: Conceptualization, Data curation, Resources, Software, Visualization, Writing – original draft. AX: Conceptualization, Data curation, Investigation, Software, Supervision, Visualization, Writing – original draft. KW: Conceptualization, Investigation, Methodology, Resources, Software, Visualization, Writing – original draft. YY: Conceptualization, Investigation, Software, Supervision, Validation, Visualization, Writing – original draft. YaS: Conceptualization, Data curation, Software, Validation, Visualization, Writing – review & editing. YoS: Conceptualization, Data curation, Funding acquisition, Project administration, Software, Supervision, Validation, Visualization, Writing – review & editing.
