# Supplementary material for: The efficacy of repetitive transcranial magnetic stimulation in postherpetic neuralgia: a meta-analysis of randomized controlled trials
Source: Front Neurol. 2024 Jun 11;15:1365445. doi: 10.3389/fneur.2024.1365445 (PMC11196813; doi:10.3389/fneur.2024.1365445)
Supplement: Supplementary file 5 [file Table_1.DOCX]

Supplementary Table 1 Search strategies in PubMed, Embase, China National Knowledge Infrastructure (CNKI), and WANFANG DATA databases.

| Database | Search strategy |
| --- | --- |
| PubMed | ("Transcranial Magnetic Stimulation"[Mesh] OR "Transcranial Magnetic Stimulation" OR "TMS" OR "rTMS") AND ("Neuralgia, Postherpetic"[Mesh] OR "Postherpetic Neuralgia" OR "Herpetic Neuralgia" OR "PHN") |
| Embase | ('transcranial magnetic stimulation'/exp OR 'Transcranial Magnetic Stimulation' OR 'TMS' OR 'rTMS') AND ('postherpetic neuralgia'/exp OR 'Postherpetic Neuralgia' OR 'Herpetic Neuralgia' OR 'PHN') |
| CNKI | (TKA=重复经颅磁刺激 OR TKA=经颅磁刺激 OR TKA=Transcranial Magnetic Stimulation OR TKA=TMS OR TKA=rTMS) AND (TKA=带状疱疹 OR TKA=Postherpetic Neuralgia OR TKA=Herpetic Neuralgia OR TKA=PHN) |
| WANFANG DATA | THEME=(经颅磁刺激 OR 重复经颅磁刺激 OR Transcranial Magnetic Stimulation OR TMS OR rTMS) AND THEME=(带状疱疹 OR Postherpetic Neuralgia OR Herpetic Neuralgia OR PHN) |
