# Supplementary material for: The efficacy of repetitive transcranial magnetic stimulation in postherpetic neuralgia: a meta-analysis of randomized controlled trials
Source: Front Neurol. 2024 Jun 11;15:1365445. doi: 10.3389/fneur.2024.1365445 (PMC11196813; doi:10.3389/fneur.2024.1365445)
Supplement: Supplementary file 8 [file Table_4.DOCX]

| VAS at 3 months post-treatment | I^2^ |
| --- | --- |
| Omitting Wang et al. 2023 | 95.63% |
| Omitting Pei et al. 2019 | 93.70% |
| Omitting Ma et al. 2015 | 95.76% |
| Omitting Pu et al. 2017 | 93.56% |
| Omitting Chen et al. 2021 | 93.13% |

Supplementary Table 4 Sensitivity analysis of the VAS at 3 months post-treatment.
