# Supplementary material for: The efficacy of repetitive transcranial magnetic stimulation in postherpetic neuralgia: a meta-analysis of randomized controlled trials
Source: Front Neurol. 2024 Jun 11;15:1365445. doi: 10.3389/fneur.2024.1365445 (PMC11196813; doi:10.3389/fneur.2024.1365445)
Supplement: Supplementary file 10 [file Table_6.DOCX]

| SF-MPQ | I^2^ |
| --- | --- |
| Omitting Wang et al. 2023 | 93.95% |
| Omitting Pei et al. 2019 | 82.45% |
| Omitting Ma et al. 2015 | 94.45% |
| Omitting Pu et al. 2017 | 84.99% |
| Omitting Chen et al. 2021 | NA |

Supplementary Table 6 Sensitivity analysis of the SF-MPQ.
